# 中国肺癌和烟草流行及控烟现状

**DOI:** 10.3779/j.issn.1009-3419.2017.08.01

**Published:** 2017-08-20

**Authors:** 小农 邹, 漫漫 贾, 鑫 王, 修益 支

**Affiliations:** 1 100021 北京，国家癌症中心/中国医学科学院北京协和医学院肿瘤医院，全国肿瘤防治研究办公室 National Cancer Center/Cancer Hospital, Chinese Academy of Medical Sciences and Peking Union Medical College, Beijing 100021, China; 2 100053 北京，首都医科大学宣武医院胸外科 Xuanwu Hospital, Capital Medical University, Beijing 100053, China

**Keywords:** 肺肿瘤, 吸烟, 二手烟暴露, 控烟, Lung neoplasms, Smoking, Secondhand smoking, Tobacco control

## Abstract

肺癌居我国恶性肿瘤死亡和发病的首位。肺癌与吸烟和二手烟暴露密切相关。近年来，我国采取了一系列控烟和肺癌防治措施，但肺癌负担依然严重，男性吸烟率仍居高不下，非吸烟者二手烟暴露十分广泛。本文对我国近年来肺癌、吸烟和二手烟暴露的流行现状进行描述，同时对我国目前所实施的控烟措施进行了综述。

## Abstract (en)

Lung cancer is the leading cause of cancer death and morbidity in China. Smoking and exposure to secondhand smoking closely related to lung cancer. Recently, series policies of tobacco control and lung cancer prevention was carried out. However, burden of lung cancer is still serious, and smoking rate in male is still very high, and in never smokers exposure to secondhand smoking is still very extensive. In this paper, epidemic situation of lung cancer, smoking and second hand smoking are described, at the same time, current implementations of tobacco control policies are summarized.

原发性支气管肺癌（肺癌），是来源于气管支气管粘膜或腺体的恶性肿瘤，主要组织类型有鳞癌、腺癌、小细胞癌和大细胞癌^[1]^，是全球常见的恶性肿瘤之一。Globocan 2012显示，我国肺癌发病人数占全球恶性肿瘤死亡的35.78%，死亡占全球的37.55%^[2]^。肺癌的发生与许多因素有关，如吸烟和二手烟，二氧化硅、石棉、锡、煤焦等职业因素，饮食，烹饪油烟，空气污染等^[3-5]^。吸烟和二手烟暴露均是肺癌的重要危险因素，其中吸烟可增加3倍以上的肺癌发生风险^[6, 7]^。我国72.4%非吸烟者暴露于二手烟^[8]^，暴露广泛，对非吸烟者肺癌发生的影响不可忽略。本文将对我国肺癌、吸烟和二手烟暴露的流行现状进行简要综述。

## 1 肺癌流行现状

### 1.1 肺癌发病和死亡现状

2013年全国癌症登记中心数据显示，中国肺癌发病73.3万人，死亡59.1万人，发病率和死亡率分别为53.86和43.41（发病率和死亡率的单位均为1/10万，以下均省略该单位），世界人口年龄标化（世标）后分别为36.09和28.41，均位居我国恶性肿瘤发病和死亡首位。男性人群世标发病率和世标死亡率约是女性的2倍，城市高于农村^[9]^。

肺癌发病率和死亡率在40岁及以上迅速上升，均在80岁-84岁达到高峰^[10]^。60岁以上老年人发病和死亡水平远高于全国，位居老年人群中恶性肿瘤发病和死亡的首位（表 1）^[11]^。

**1:** 2013年中国肺癌的发病和死亡情况（1/10^5^） Incidence and mortality of lung cancer in China, 2013 (1/10^5^)

	Crude incidence	ASIRW	Crude mortality	ASMRW
Total	53.86	36.09	43.41	28.41
Gender				
Male	70.10	49.62	57.64	40.30
Female	36.78	23.18	28.45	17.21
Area				
Urban	54.53	35.48	44.38	36.83
Rural	53.08	28.09	42.27	28.77
≥60 years	251.64	239.03	218.18	200.98
ASIRW: age-standardized incidence rate by world population; ASMRW: age-standardized mortality rate by world population.				

### 1.2 肺癌发病和死亡趋势

前三次死因调查数据显示，肺癌世标死亡率居第一位，从1973年-1975年的7.30迅速上升至2004年-2005年27.62，是我国人群死亡率上升最快的癌种；男性从10.10上升至39.06，女性从4.70上升至16.73，两者上升速度相似^[12, 13]^。22个登记处数据显示，2000年-2011年，男性肺癌世标发病率每年下降0.2%，女性则以0.9%速度上升^[14]^。我国男女和城乡的肺癌发病水平差异明显缩小，发病年龄趋于老龄化：1989年-2008年男女肺癌发病比由2.47降至2.28，城乡由2.07降至1.14，男性平均发病年龄由65.32岁上升至67.87岁，女性由65.14岁升高至68.05岁^[15]^。

经济发达的北京市和上海市，肺癌粗发病率和死亡率均呈上升趋势^[16, 17]^，排除年龄影响后北京市肺癌中标发病率从1988年20.62变化至2007年21.48，死亡从18.19变化至17.26，变化不大；上海市肺癌中标发病率从25.28至20.29；死亡从21.34至16.87，略呈下降趋势^[10]^。在大连，女性世标发病率从1991年-1995年23.7至2006年-2010年31.1显著上升，男性则趋于稳定（55.1、57.9）^[18]^。以上数据说明近年来，一些大城市肺癌发病和死亡水平的增高主要受年龄影响，但由于其中女性肺癌受一些特定危险因素如二手烟、烹饪油烟等的影响较大，发病率呈上升趋势。在经济欠发达的江苏省启东市，肺癌世标发病率从1972年14.42升高至2001年36.44^[19]^，死亡从1972年10.13上升至2008年-2011年29.50^[20]^，上升趋势明显。云南省个旧市和宣威市是肺癌高发区，个旧以矿工为高发人群，1996年-2005年中标死亡率从54.91上升至70.58^[21]^；宣威农民高发，中标死亡率从1973年-1975年28.14增至2003年-2005年83.16^[22, 23]^，远高于同期全国水平。提示排除年龄影响后，一些经济欠发达地区的肺癌发生和死亡受特定危险因素的影响较大。

### 1.3 肺癌病理类型流行现状和趋势

肺癌病理类型主要有非小细胞肺癌和小细胞肺癌，前者又分为鳞癌、腺癌、大细胞癌等，分布与吸烟情况密切相关，吸烟者病理类型以鳞癌，小细胞肺癌为主，非吸烟者则以腺癌为主^[13]^。国外研究^[24, 25]^报道，近年来肺癌病理类型以腺癌的上升和鳞癌的下降为主要趋势，中国也呈现相似趋势。邹小农等^[26]^对北京市某医院2000年-2012年间15, 427例男性肺癌患者分析，腺癌构成比从21.96%上升至43.36%，鳞癌从39.11%下降至32.23%，变化显著；7, 070例女性肺鳞癌所占比例较低，从15.69%降至5.97%，腺癌从46.72%上升至76.49%^[27]^。徐小熊等^[28]^对上海市某医院20年间12, 117例肺癌患者研究也表明，男性患者中鳞癌多见，且呈显著下降趋势，女性腺癌最为常见且上升迅速。宁夏和郑州地区的医院人群研究^[29, 30]^也显示，肺鳞癌患者比例下降，腺癌上升。多项研究^[31-33]^称，这种改变可能是由于烟草成分中“低焦油”和“过滤香烟”的使用导致烟草成分更容易到达肺外周和肺泡。

## 2 吸烟和二手烟流行现状

### 2.1 吸烟和二手烟现状

吸烟可导致多种肿瘤的发生，如肺癌、口腔和鼻咽癌、食管癌、乳腺癌、结直肠癌等，其与肺癌的关系尤为密切^[34]^。2015年成人烟草调查报告指出，我国15岁以上成人现在吸烟率为27.7%，男性52.1%，女性2.7%。农村男性现在吸烟率（55.4%）高于城市（49.0%），女性则差异不大（2.8%、2.7%）。男性和女性大专以上者的现在吸烟率（41.9%、0.9%）均低于初中水平者（61.3%、4.8%）。男性职业人群中，务农者吸烟比例最高60.0%，学生最低为10.9%，医务人员和教师分别为43.0%和48.1%。电子烟在我国使用率较低，男性5.6%，女性0.6%^[35]^。青少年中的烟草使用情况也不容乐观。31个省市155, 117名初中生调查显示，初中男生现在烟草使用率高达11.2%，西藏、云南、贵州甚至高达37.2%、27.0%、26.5%，尝试率男生（30.1%）高于女性（8.7%）^[36]^。

二手烟暴露没有安全水平，可导致肺癌^[34]^。2015年调查显示，餐馆二手烟暴露率最高，为76.3%，工作场所为54.3%，家庭57.1%，政府大楼为38.1%，医疗机构26.9%，中小学校园17.2%，公共交通工具最低为16.4%。青少年二手烟暴露现状也十分严峻。2014年青少年调查报告，过去7 d内，72.9%的青少年暴露于二手烟，男生（75.7%）高于女生（69.9%）；城乡（74.4%、72.4%）差异无统计学意义。暴露场所以室外公共场所比例最高（58.3%），其次是室内公共场所（57.2%）、家庭（44.4%）；父母和朋友吸烟的青少年更容易发生二手烟暴露^[37]^。

### 2.2 吸烟和二手烟的流行趋势

近20年间我国成年男性吸烟率仍居高不下。尽管从1996年63.0%降至2015年52.1%，仍维持在高水平，远高于世界（36.1%）和欧洲地区水平（39.0%）；女性吸烟率从1996年3.8%变化至2.7%，一直处于低水平状态（图 1）^[38]^。各地区男性吸烟率差别不大，女性以东北和华北地区较高（4.71%-17.32%）。不同地区青少年烟草使用流行趋势不同，段佳丽等对北京市2008年-2014年177所学校调查显示，大、中学生尝试吸烟率从30.05降至24.30%^[39]^，广州市青少年健康危险行为监测资料显示，青少年尝试吸烟从2008年26.61%降至2013年22.44%^[40]^，而江苏省从2005年22.6%上升至29.6%^[41]^。关于二手烟的调查，1996年和2002年调查时采用定义相同，二手烟暴露率分别为53.84%和51.9%，变化不大，暴露场所以家庭为主，公共场所和工作场所暴露比例较低；2010年与2015年采用定义相同，未对二手烟暴露时间进行限定，2010年暴露率为72.4%，暴露场所以公共场所为主，2015年调查也显示暴露场所以公共场所最高，与2010年相比各场所的暴露率均有所改善（图 2）^[8, 35, 42, 43]^。

**1:**
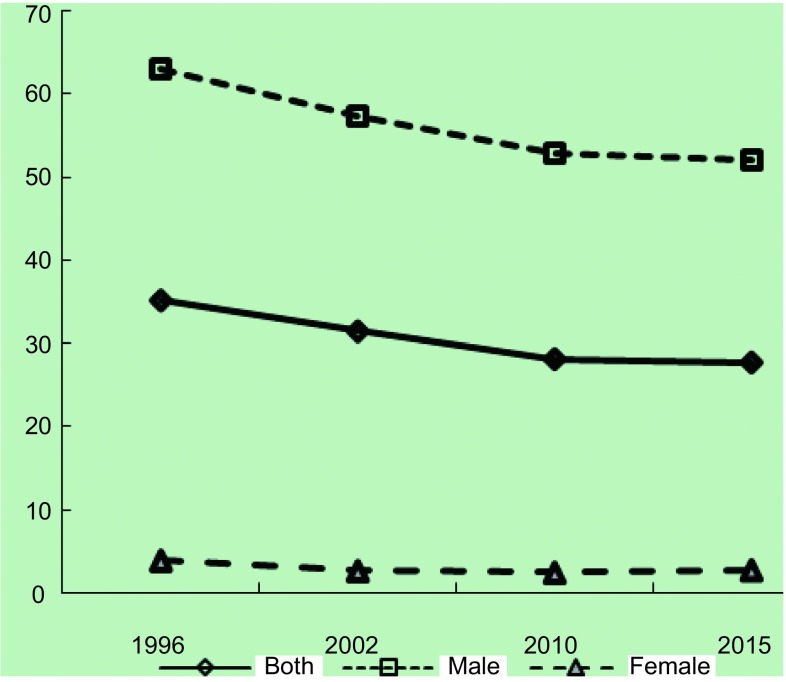
我国成人现在吸烟率变化 Trends of current smoking in adult, China

**2:**
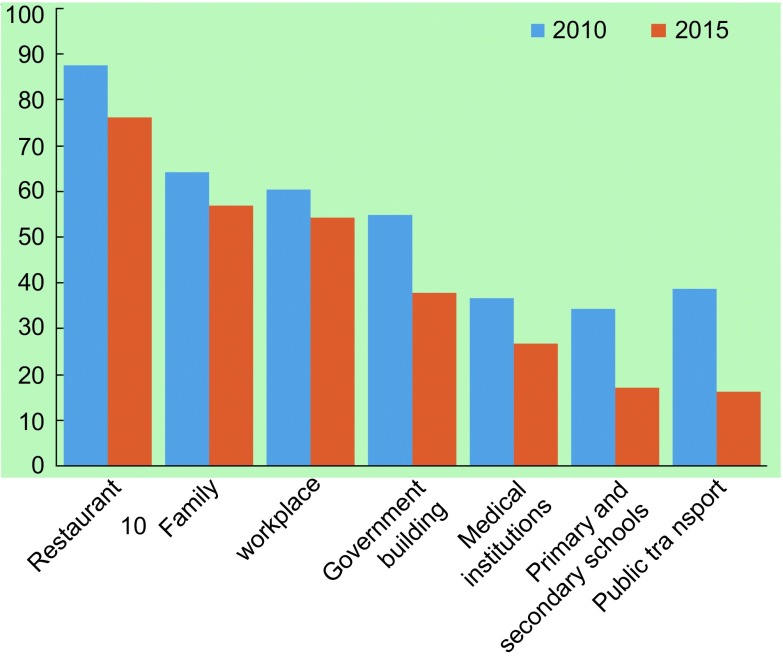
2010年和2015年各场所看的有人吸烟的百分比 Percentage of people smoking in different places, in 2010 and 2015

### 2.3 控烟措施

世界卫生组织（World Health Organization, WHO）提出最有效的控烟措施为以下六种：M：监测烟草流行和预防政策；P：保护人们免受烟草烟雾危害；O：提供戒烟帮助；W：警示烟草危害；E：确保禁止烟草广告、促销和赞助；R：提高烟税。

自从加入《烟草控制框架公约》，我国为控制烟草流行采取了一系列措施。在烟草流行监测方面，我国已开展5次全国范围内成人烟草调查，并于2014年开展了第一次全国青少年烟草调查。国际研究^[44]^提示，降低烟草危害最有效的办法是控烟立法。目前，北京、天津等18个城市制定了地方性控烟立法，其中北京《控烟条例》被誉为史上最为严厉的控烟法规，明确规定所有室内公共场所和工作场所为禁烟区^[45]^，但仍未出台国家层面的控烟法。中国吸烟者的戒烟比（14.4%）在世界上处于较低水平^[35]^。戒烟不仅需要自身决定还需要周围环境尤其是医务人员的正确引导。调查^[8, 45]^显示我国医务人员对于戒烟者提供的戒烟帮助较少，使用药物及咨询戒烟比例为3.1%、3.0%，90%以上戒烟者误认为戒烟只要下定决心就能成功。

上海、北京、郑州等校园控烟教育和环境干预后，青少年尝试吸烟率下降3.1%，实际吸烟率下降1.2%；教师对吸烟与脉管炎和男性性功能障碍相关的知晓率由58.27%、66.71%分别上升至71.10%、78.30%，现在吸烟率由56.99%降至36.80%，均有积极作用^[46, 47]^。我国青少年最常接触烟草广告的渠道是电视（21.3%），烟草零售点的广告和促销现状较为严重（41.3%）^[48]^，需进一步落实全面禁止烟草广告、促销和赞助的《广告法》实施。提高烟草价格和税率是最有效的降低烟草流行措施。我国于2009年提高烟草税收，但2007年卷烟平均价格6.26元与2010年6.28元相比几乎没有变动^[49]^；2010年-2015年城市居民卷烟费用占人均可支配的收入比重由10.5%降至8.8%，农村从21.1%降至17.3%，烟草相对消费价格降低^[50]^。2015年5月10日起我国再次提高烟草税收，从5%提升至11%，但孟加拉国，新西兰等国家已升至75%以上，我国烟草税收依然处于较低水平^[51]^。提高烟草税收，尤其提高烟草最低价格，降低低收入人群的卷烟消费量，不仅可降低烟草流行，而且能有效防止青少年吸烟。

## 3 结语

肺癌位居我国恶性肿瘤发病和死亡的首位，经济发达地区的肺癌发病和死亡率趋于稳定，欠发达地区则逐年增长，城乡和男女差距逐渐缩小。肺癌的发生和死亡与吸烟和二手烟暴露密切相关。目前我国男性吸烟率仍居高不下，非吸烟者二手烟暴露仍十分广泛，虽然我国实施了一系列控制烟草流行的措施，但效果仍不显著，亟需实施系统的具有中国特色的控烟措施。控制烟草流行，降低肺癌的发生和死亡，仍任重道远。
